# Orthogonally conjugated phthalocyanine-porphyrin oligomer for NIR photothermal-photodynamic antibacterial treatment

**DOI:** 10.1038/s42004-025-01470-w

**Published:** 2025-03-14

**Authors:** Guixue Lian, Wanru Zhao, Gaoqiang Ma, Sen Zhang, Ailin Wu, Lin Wang, Dongjiao Zhang, Wei Liu, Jianzhuang Jiang

**Affiliations:** 1 School of Crystal Materials, State Key Laboratory of Crystal Materials, Shandong University, Jinan, 250100 China; 2 Department of Implantology, School and Hospital of Stomatology, Cheeloo College of Medicine, Shandong University, Jinan, 250100 China; 3 Shandong Key Laboratory of Oral Tissue Regeneration & Shandong Engineering Laboratory for Dental Materials and Oral Tissue Regeneration & Shandong Provincial Clinical Research Center for Oral Diseases, Jinan, 250100 China; 4 Department of Chemistry and Chemical Engineering, School of Chemistry and Biological Engineering, University of Science and Technology Beijing, Beijing, 100083 China; 5 Beijing Key Laboratory for Science and Application of Functional Molecular and Crystalline Materials, Beijing, 100083 China; 6 Beijing Advanced Innovation Center for Materials Genome Engineering, Beijing, 100083 China

**Keywords:** Drug discovery and development, Nanoparticle synthesis, Photobiology

## Abstract

With the increase of antibiotic resistance worldwide, there is an urgent demand to develop new fungicides and approaches to address the threat to human health posed by the ineffectiveness of traditional antibiotics. In this work, an orthogonal conjugated uniform oligomer bactericide of SiPc-ddCPP was constructed between silicon phthalocyanine and porphyrin, which can effectively treat infection through photodynamic-photothermal combined therapy without considering drug resistance. Compared with organic photothermal agents induced by unstable *H*-aggregation with blue-shifted absorption and fluorescence/ROS quenching, this orthogonal-structured uniform SiPc-ddCPP nanoparticle shows remarkably stability and NIR photothermal effect *(η* = *31.15%)* along with fluorescence and ROS generation. Antibacterial studies have shown that both Gram-positive and Gram-negative bacteria could be efficiently annihilated in a few minutes through synergistic PDT-PTT along with satisfactory bacterial targeting. These results suggest SiPc-ddCPP is a multifunctional NIR bactericide, which afford a new approach of synergistic PDT-PTT sterilization to conquer the crisis of antibiotic resistance.

## Abstract (web summary)

With the increase in antibiotic resistance worldwide, light-induced phototherapies, including photodynamic and photothermal therapy, have attracted interest due to their unique mode of action. Here, the authors report an orthogonally conjugated oligomer bactericide of SiPc-ddCPP, constructed between a silicon phthalocyanine and a porphyrin *via* an amide bond, and show that it can effectively treat infection through combined photodynamic–photothermal therapy without needing to consider drug resistance.

## Introduction

The rapid growth of antibiotic resistance has seriously threatened human health worldwide due to the overuse of antibiotic in public health and food industries^1–3^. Over the past few decades, conventional antibiotics have shown growing ineffectiveness in treating some infections in hospitals^4^. For example, as reported by US National Institutes of Health, the number of methicillin-resistant Staphylococcus aureus strains (t002) has increased significantly since 2013^5^. However, antibiotics are still the primary clinical treatment for bacterial infections. Furthermore, the shortage of novel antibiotics is exacerbating these public health issues^4,6,7^. Therefore, the development of new antibacterial agents and methods with high efficiency, safety and convenience has been urgently put on the agenda^8–12^.

Light-induced phototherapies, including photodynamic therapy (PDT) and photothermal therapy (PTT), have attracted more and more interest in anticancer and antibacterial treatment due to their unique noninvasiveness, minimal side effects and wide range of adaptability^13–19^. Comparing with conventional antibiotic treatment, both PDT and PTT enable repetitive treatments without dosage limitation and risk of drug resistance, although they are based on different therapeutic mechanisms. In PDT, highly reactive oxygen species (ROS) were produced by photon excited photosensitizer (PS) to exterminate bacteria^20–23^; In PTT, light triggers the photothermic agent (PTA) to produce heat, which then induces damage or death of bacteria^24–27^. Its oxygen-independent nature endows it with decent therapeutic efficacy against anaerobic bacteria in hypoxia environment (Supplementary Fig. 1). The combined use of PDT and PTT for cancer treatment has shown enormously enhanced therapeutic efficacy^28–32^, which is not only due to the therapeutic effect superposition of PDT and PTT, but also PTT heat can improve oxygen concentration through accelerating blood flow to make up the oxygen consumption of PDT. In term of the similar replication rate and highly uptake capability of cancerous cells and bacteria^33^, PDT and PTT have the same therapeutic effect on them. Whereas, the antibacterial studies on either PDT or PTT are far behind that for anticancer treatment. In particular, the combined PDT-PTT antibacterial studies have been rarely reported.

So far, various materials have been developed for PDT and PTT, such as carbon-based nanoparticles^34,35^, gold nanoparticles^36–38^, metal compounds^39–41^ and organic dyes^42–44^, etc. Among these materials, porphyrin (Por)^45,46^ and phthalocyanine (Pc)^47–49^ have been extensively studied as either photosensitizer or photothermal agent based on different mechanisms, because of their extraordinary photophysical and photochemical properties as well as excellent stabilities and low toxicity. Compare to inorganic materials with long-term toxicity and low biocompatibility, Por is a class of natural compound that widely exist in organisms, participating in important biochemical processes of photosynthesis and oxygen-transport, which endow it with unique affinity for bacteria and cancerous cells^50^; On the other hand, Pc is the synthetic analog of Por, which shows much higher absorption than Por in red to NIR region that is considered as optimal therapeutic window for treatment deep-seated lesions^51^. In addition, silicon phthalocyanine (SiPc)^52^ with two axial ligands on the opposite sides of the Pc planar is recognized to be one of efficient PS for PDT because of its high ROS generation and excellent biocompatibility, of which the axial ligands can tremendously break the *π-π* interaction to enhance its solubility and photoactivity. In fact, photothermal effect of Por and Pc are mainly based on *H*-aggregation induced non-radiative vibration relaxation, however, at the expense of blue-shifted absorption, quenching of both fluorescence and PDT^53–55^ (Fig. S1). Accordingly, how to construct Pc and Por to maximize synergistic PDT-PTT therapeutic effect is of great challenge for researchers.

**Fig. 1:**
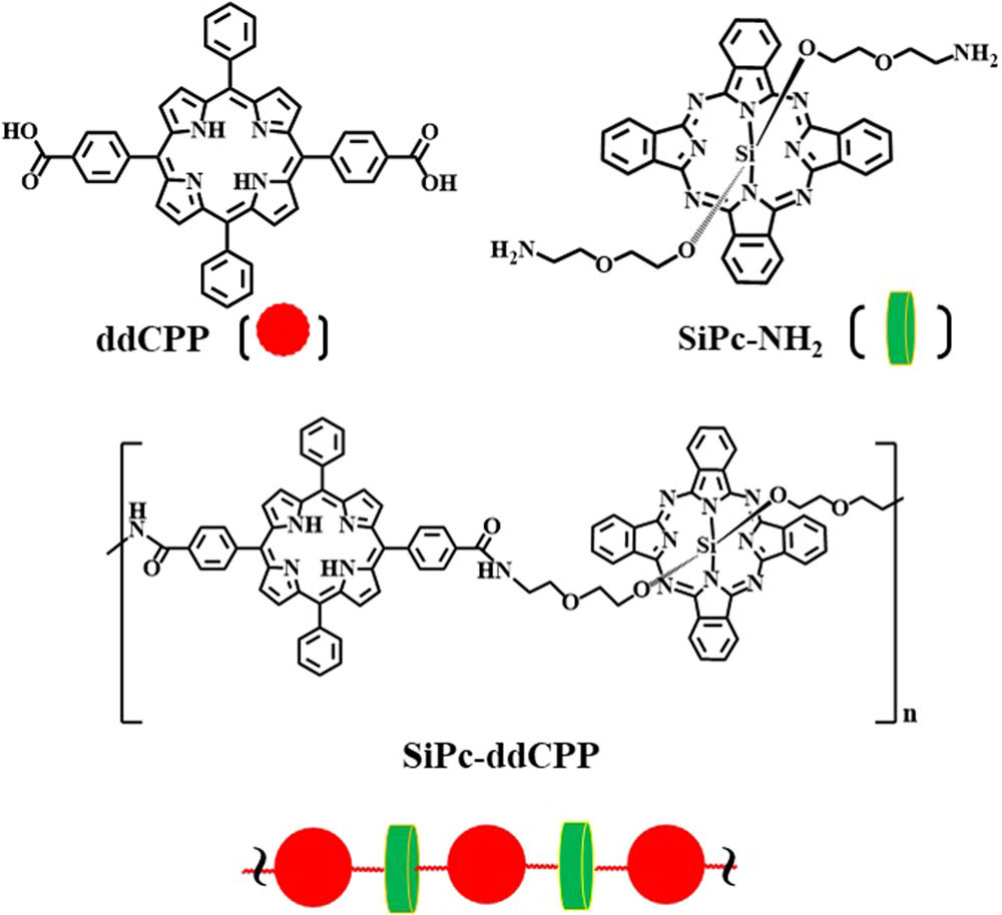
Molecular structure. Chemical structure of 5,15-diphenyl-10,20-di(4-carboxyphenyl)porphine (ddCPP), silicon phthalocyanine (SiPc-NH_2_) and orthogonal conjugate SiPc-ddCPP.

In the present work, an orthogonal conjugated oligomer between Por and Pc was design and constructed, starting from silicon phthalocyanine (SiPc-NH_2_) and 5,15-diphenyl-10,20-di(4-carboxyphenyl)porphine (ddCPP). The conjugation reaction between axial -NH_2_ and peripheral -COOH results in orthogonal structure between phthalocyanine and porphyrin (Fig. 1). The obtained SiPc-ddCPP exhibits good solubility both in organic and aqueous solvents due to the suppression of π-π aggregation, and remarkable NIR photothermal effect (*η* = 31.15%) accompanied with fluorescence emission and ROS generation. Antibacterial studies indicated that SiPc-ddCPP show bacterial-affiliated synergistic PDT-PTT therapeutic effect on both Gram-positive and Gram-negative anaerobic bacteria. These results suggest SiPc-ddCPP is a novel multifunctional NIR bactericide for PDT-PTT synergistic sterilization of both anaerobic and aerophile bacteria.

## Results and discussion

### Synthesis and characterization of SiPc-ddCPP

**Scheme 1:**
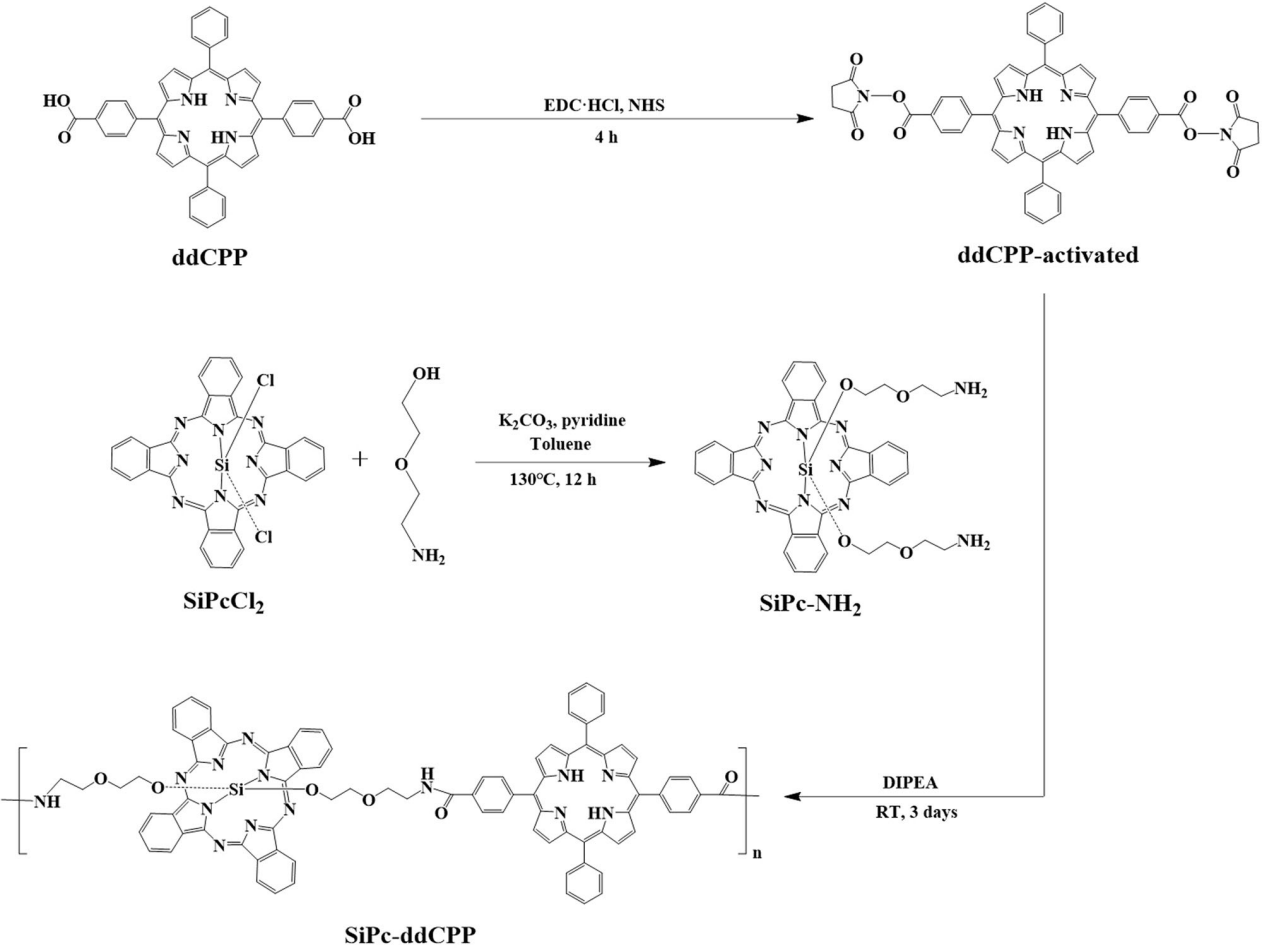
Synthesis. Synthetic procedure of SiPc-ddCPP.

As a macrocyclic conjugated planar architecture, phthalocyanine tend to employ face-to-face *H*-aggregation. Although the *H*-aggregates favor photothermal effect through non-radiative vibration relaxation, they are unstable in physiological environment due to the weak *π-π* interaction within the aggregate, leading to reduced PTT therapeutic efficiency. In addition, *H*-aggregation will induce blue-shifted absorption and quenching of both fluorescence and ROS generation, which not only inhibits the PDT effect but also weaken the tissue penetration by light. This competition nature between PDT and PTT greatly hinders the realization of their synergistic therapeutic effect. Our recent studies on phthalocyanine based photothermal materials indicated that the intramolecular energy transfer between different chromophores could significantly increase photothermal effect without quenching of fluorescence^56^. Herein, to achieve combined PDT-PTT effect, amide-bond connected hybrid oligomer (SiPc-ddCPP) featuring orthogonal configuration between silicon phthalocyanine and porphyrin was constructed with expectation of enhanced PTT effect without quenching of PDT and fluorescence. Firstly, starting material of SiPc-NH_2_ was synthesized by substitution of SiPcCl_2_ with 2-(2 aminoethoxy) ethanol using previously reported method^57,58^. Subsequently, SiPc-ddCPP was constructed between SiPc-NH_2_ and ddCPP through coupling of -NH_2_ and -COOH by EDC/NHS conjugation procedure with the SiPc-NH_2_/ddCPP molar ratio of 2:1. The reaction mixture was successively purified by dialysis in DMF and H_2_O (molecular weight cutoff: 1000) to remove unreactive starting materials and conjugation reagents. Finally, SiPc-ddCPP was obtained by freeze-drying as dark powder (Scheme 1). It worth to mention, after several attempts, optimal particle size of SiPc-ddCPP (~100 nm) could be obtained at the SiPc-NH_2_/ddCPP molar ratio of 2:1, which is used for the following biological application studies.

**Fig. 2:**
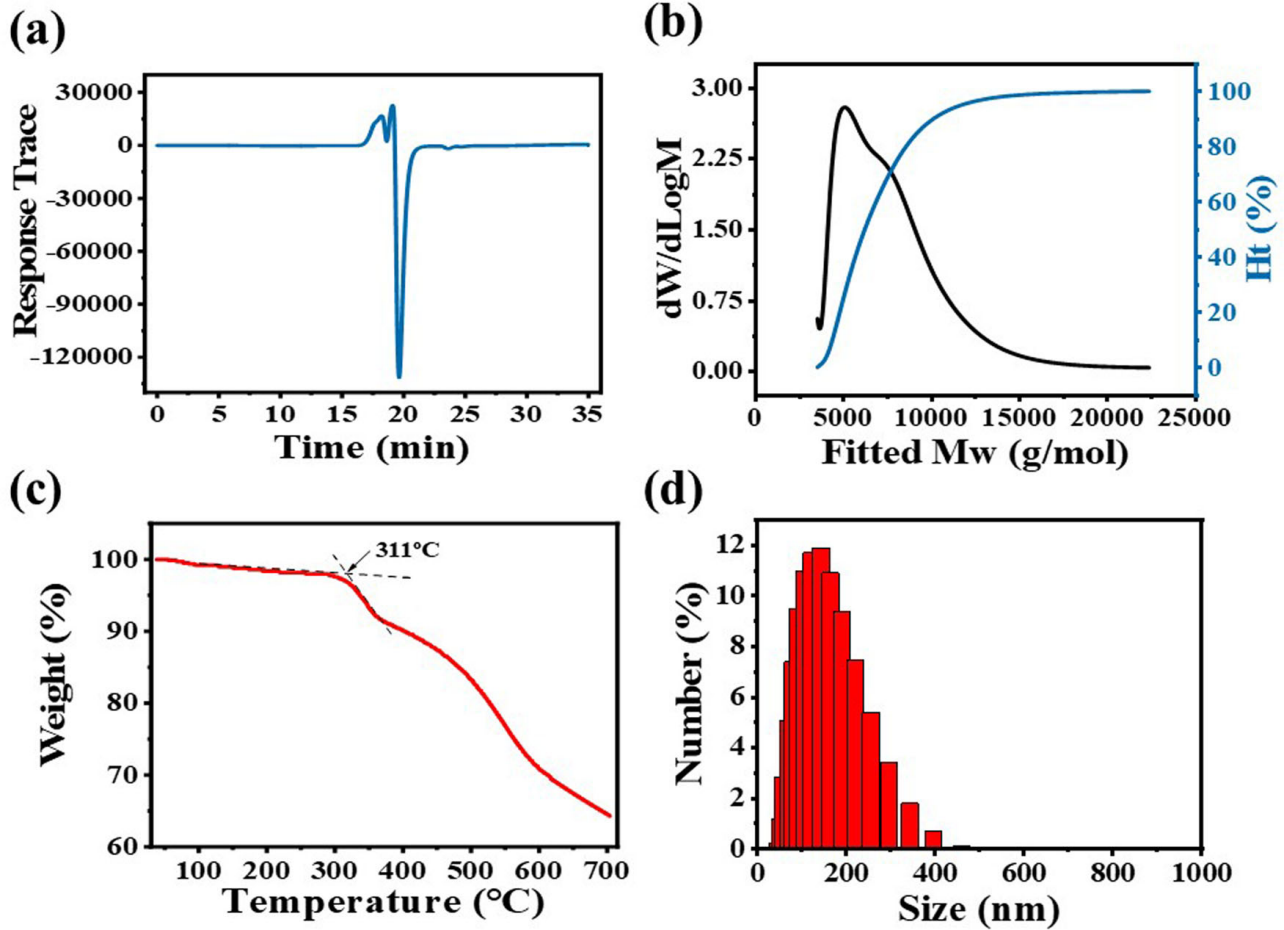
Molecular and size distribution. The (**a**) effluent curve and (**b**) molecular weight distribution map of SiPc-ddCPP in GPC; (**c**) TG curve of SiPc-ddCPP; (**d**) DLS analysis of SiPc-ddCPP in H_2_O.

Gel permeation chromatography (GPC) was used to determine the molecular weight and purity of SiPc-ddCPP. It can be seen from the outflow curve of GPC (Fig. 2a), the compounds were separated out at ~17 min, shown as a narrow sharp peak, which is corresponding to the molecular mass of Mw_max_ ~ 5200 in the molecular weight distribution curve (Fig. 2b). Accordingly, the condensation number in SiPc-ddCPP is calculated to be *n* ≈ 4. Atomic absorption spectrum (AAS), a method to qualitative and quantitative analysis of element by its characteristic absorbance, was used to further evaluate the purity of SiPc-ddCPP. The Si content was detected to be 0.2825 mg L^-1^ in SiPc-ddCPP (1 μM per unit) with a small 0.53% deviation from the theoretical value of 0.2810 mg L^-1^, showing the good purity of SiPc-ddCPP (Supplementary Fig. 2). The particle size distribution of SiPc-ddCPP in H_2_O was detected by dynamic light scattering (DLS) method. As shown in Fig. 2d, the particle size of SiPc-ddCPP is ca.100 nm, locating at the optimal particle range for biological studies. In addition, thermogravimetric analysis (TG) shows that SiPc-ddCPP start to decompose at 311 °C and exhibit the good thermostability of SiPc-ddCPP.

**Fig. 3:**
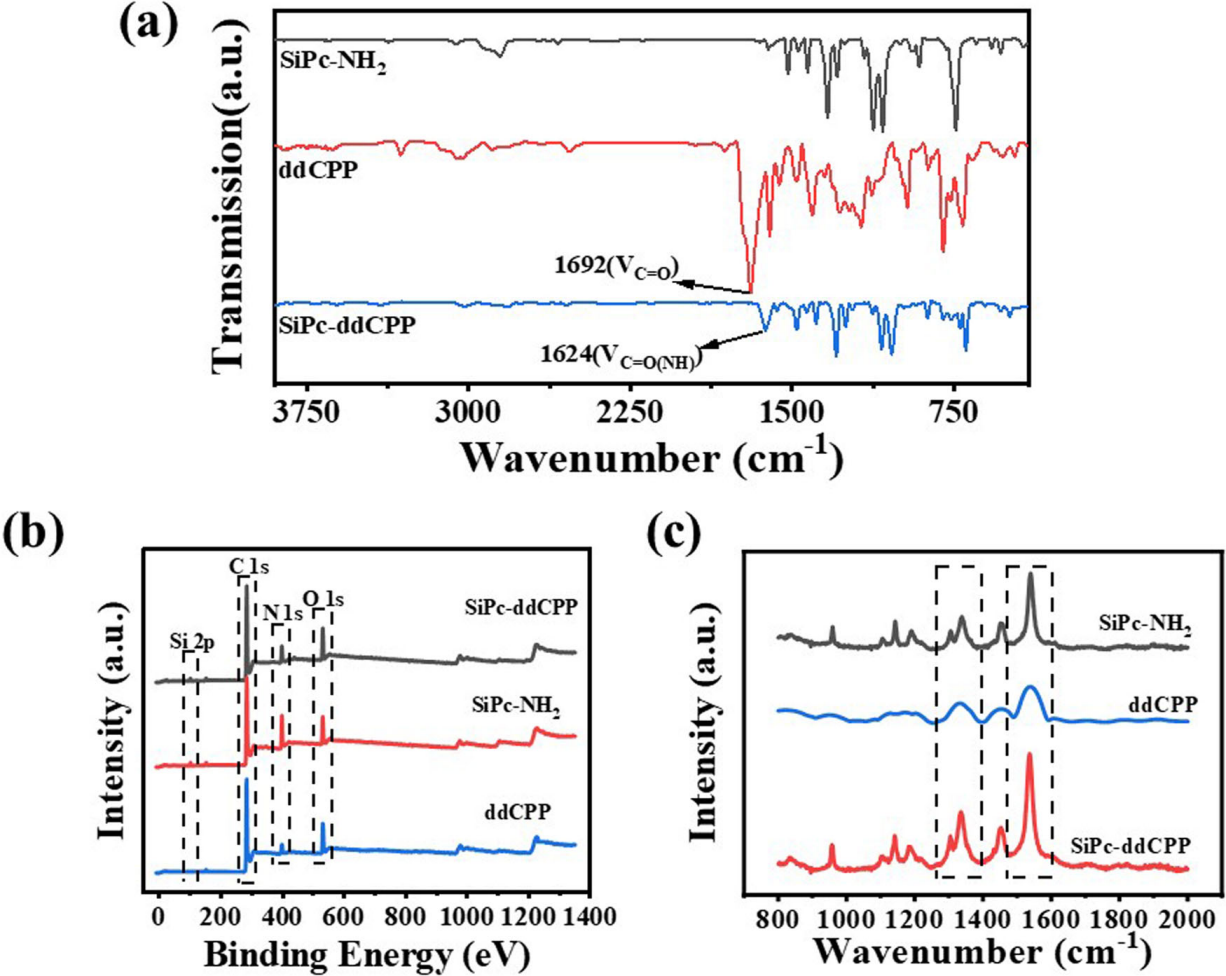
Bond formation characterization. **a** FT-IR spectra, (**b**) Roman spectra and (**c**) XPS spectra of SiPc-NH_2_, ddCPP and SiPc-ddCPP.

The bond formation was characterized by Fourier transform infrared (FT-IR) spectra, Raman spectroscopy and X-ray photoelectron spectroscopy (XPS). As shown in FT-IR spectrum of SiPc-ddCPP, the ν_C=O(OH)_ absorption at 1692 cm^-1^ from ddCPP disappeared and replaced by the absorption at 1624 cm^−1^ attributed to ν_C=O(NH)_, indicating the successful formation of amide bond between SiPc and ddCPP. The Raman spectra of SiPc-NH_2_, ddCPP and SiPc-ddCPP are compared in Fig. 3b. A superimposing of Raman signals of SiPc-NH_2_ and ddCPP was observed in SiPc-ddCPP spectrum and the weak signals of ddCPP were overlapped by that of SiPc-NH_2_, indicating the coexistence the two components. The XPS spectrum of SiPc-ddCPP also exhibit the photoelectrons of Si 2p, C 1 s, N 1 s, and O 1 s, coming from both SiPc-NH_2_ and ddCPP, further confirming the successful synthesis of the hybrid oligomer between the two components (Fig. 3).

**Fig. 4:**
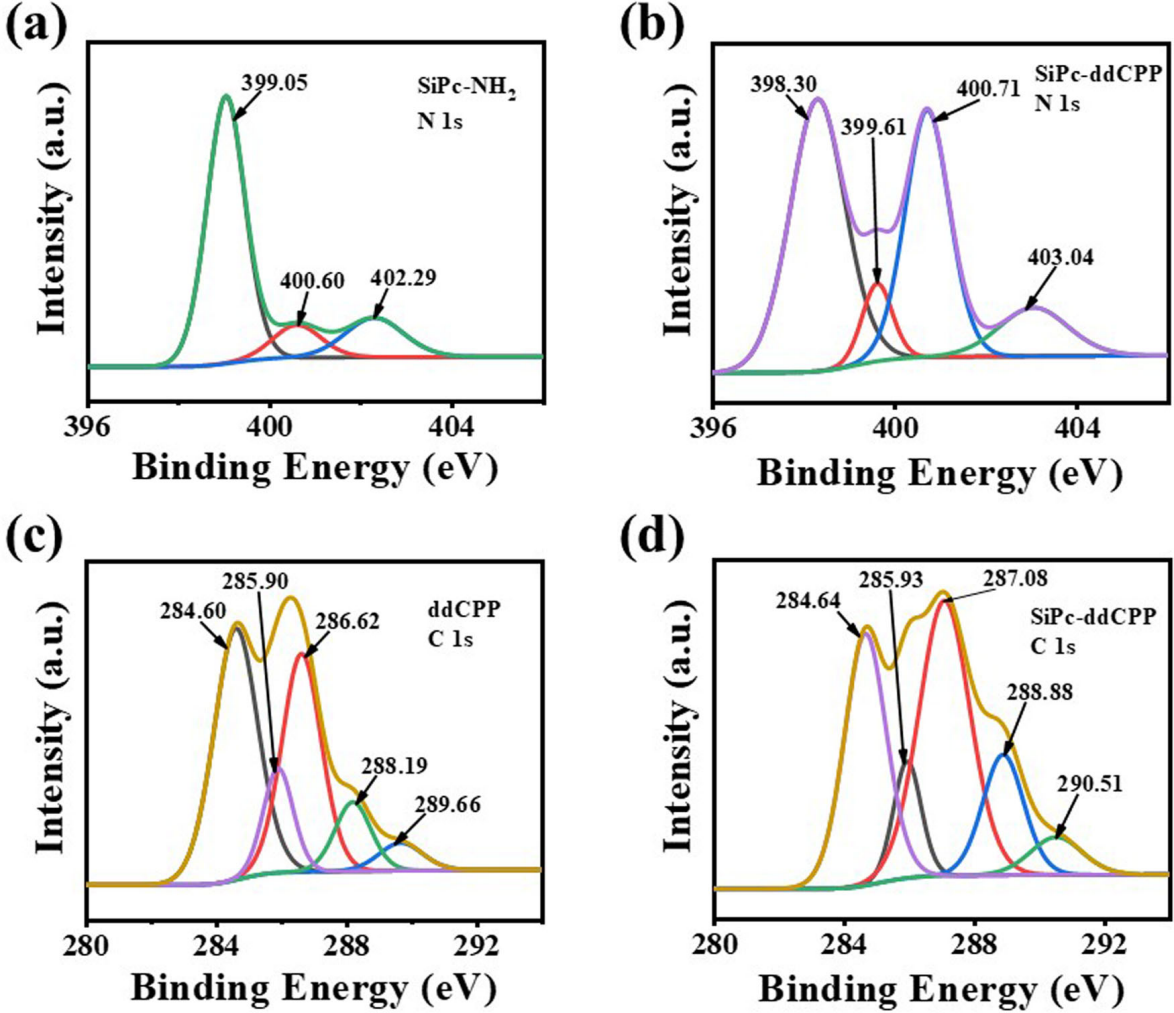
XPS analysis. N 1 s XPS spectra of (**a**) SiPc-NH_2_ and (**b**) SiPc-ddCPP; C 1 s XPS spectra of (**c**) ddCPP and (**d**) SiPc-ddCPP.

Moreover, the covalent amide bond formation was further characterized by comparison the core-level high-resolution photoelectrons spectra of C 1 s and N 1 s between SiPc-NH_2_/ddCPP and SiPc-ddCPP. The N 1 s high-resolution spectrum of SiPc-NH_2_ can be curve-fitted three peaks corresponding to nitrogen functional groups of C = N (399.05 eV), C-N (400.60 eV), and N-H (402.29 eV). For comparison, the N 1 s high-resolution spectra of SiPc-ddCPP fit four peaks corresponding to the nitrogen functional groups of C = N (398.30 eV), C-N (399.61 eV), N-H (400.71 eV) and O = C-N (403.04 eV), respectively. The appearance of new peak of O = C-N (403.04 eV) is a good proof for the formation of amide bonds. In addition, the high-resolution electronic spectrum of C 1 s were also analyzed. The C 1 s high-resolution spectrum of ddCPP was fitted with five peaks. They correspond to the carbon functional groups of C-C/C = C (284.60 eV), C-N/C = N (285.90 eV), C-O (286.62 eV), C = O (288.19 eV), O-C = O (289.66 eV), respectively. Contrastively, the C 1 s high-resolution spectrum of SiPc-ddCPP was also fitted with five peaks. They correspond to the carbon functional groups in C-C/C = C (284.64 eV), C-N/C = N (285.93 eV), C-O (287.08 eV), C = O (288.88 eV) and N-C = O (290.51 eV). The O-C = O (289.66 eV) peak belonging to ddCPP disappears and instead by the N-C = O (290.51 eV) peak of SiPc-ddCPP, which strongly proves the formation of amide bond (Fig. 4).

**Fig. 5:**
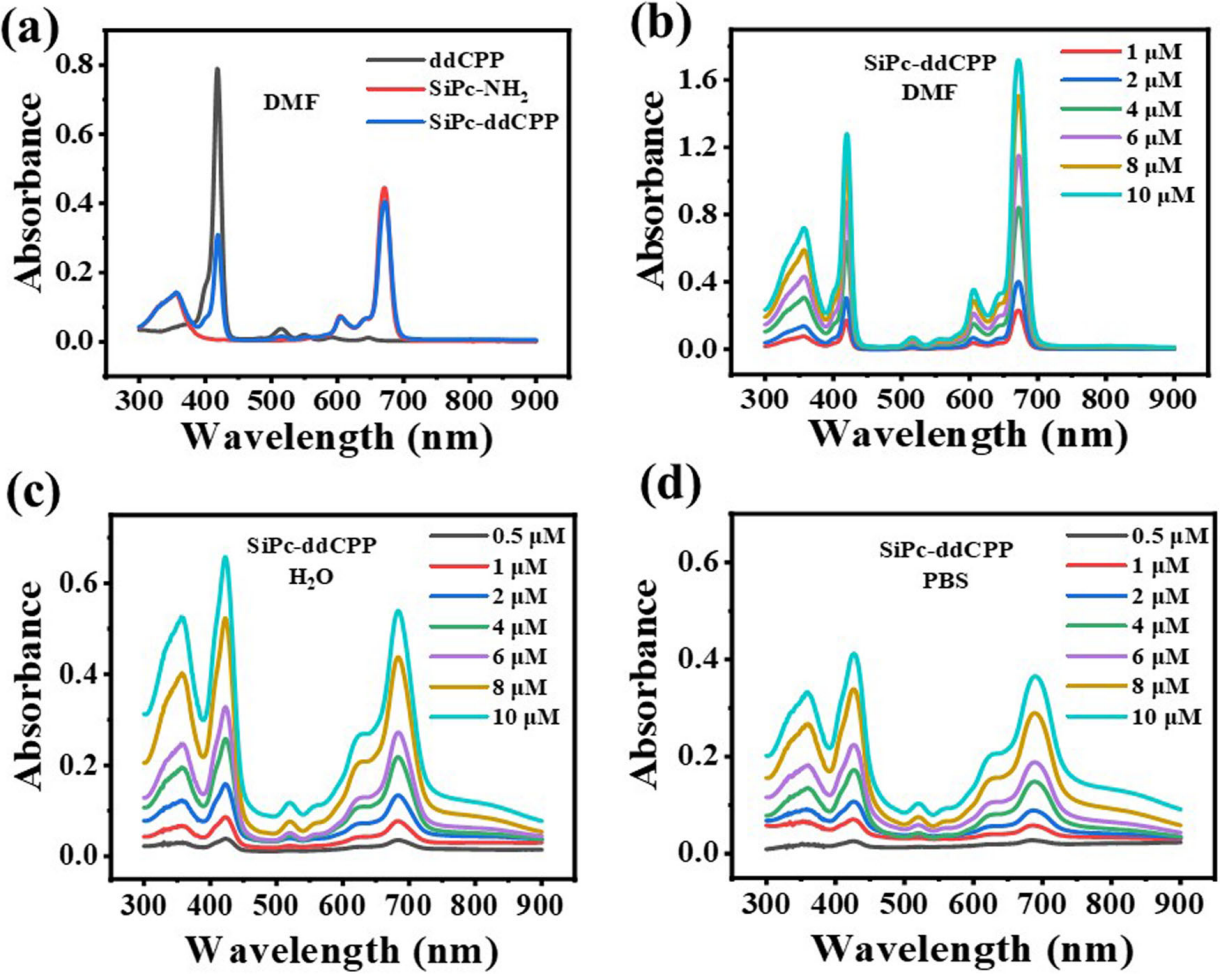
Absorption spectra. **a** UV-vis absorption of equivalent SiPc-ddCPP, ddCPP, and SiPc-NH_2_ in DMF; UV-vis absorption of SiPc-ddCPP at different concentrations in (**b**) DMF, (**c**) H_2_O and (**d**) PBS.

The fluorescence and UV-Vis absorption spectra were performed in both DMF and aqueous solution for comparison. As shown in Fig. 5, the absorption curve of SiPc-ddCPP in DMF shows the characteristic peaks from monomeric SiPc and ddCPP, indicating the coexistence of both chromophores. In addition, the absorption peaks of ddCPP fall right into the absence region of Pc, resulting in a fully absorption spectrum ranging from ultraviolet to NIR region. Due to the existence of ethylene glycol linkage chains in SiPc-ddCPP, along with its orthogonal structure, excellent dispersity in aqueous solutions was observed. The characteristic absorption of SiPc and ddCPP can be observed in H_2_O and biological PBS for SiPc-ddCPP, revealing its good solubility and the advantage of orthogonal structure of SiPc-ddCPP to prevent *π-π* aggregation in water. Comparing to the absorption in DMF, a more than 3 times higher NIR absorption can be detected due to its relatively less dispersion in aqueous environment. However, in turn, this is favorable to the NIR photothermal potential for treatment of deep-seated infections. The excitation-emission relationship of fluorescence is an indirect method to detect intramolecular energy transfer. As shown in Supplementary Fig. 3, intense fluorescence attributed to SiPc (~675 nm) could be detected for SiPc-ddCPP under excitation by either SiPc or ddCPP absorption, strongly manifesting the energy transfer from ddCPP to SiPc within SiPc-ddCPP. At equivalent concentration, the relative lower fluorescence intensity of SiPc-ddCPP than free SiPc-NH_2_ to further evidenced the intramolecular energy loss during transfer process within SiPc-ddCPP. Furthermore, both the absorption and fluorescence intensities are proportional concentration dependent, indicating the good purity and stability of SiPc-ddCPP in solution.

Time-resolved fluorescence is employed as direct method to further explore the intramolecular energy exchanges. It can be seen from Supplementary Fig. 4, the fluorescence lifetime of SiPc-NH_2_ and ddCPP are 6.59 ns and 11.05 ns, respectively, while the fluorescence lifetime of SiPc-ddCPP is significantly reduced to 2.95 ns, due to the energy transfer between the two chromophores.

### Singlet oxygen (^1^O_2_) generation

**Fig. 6:**
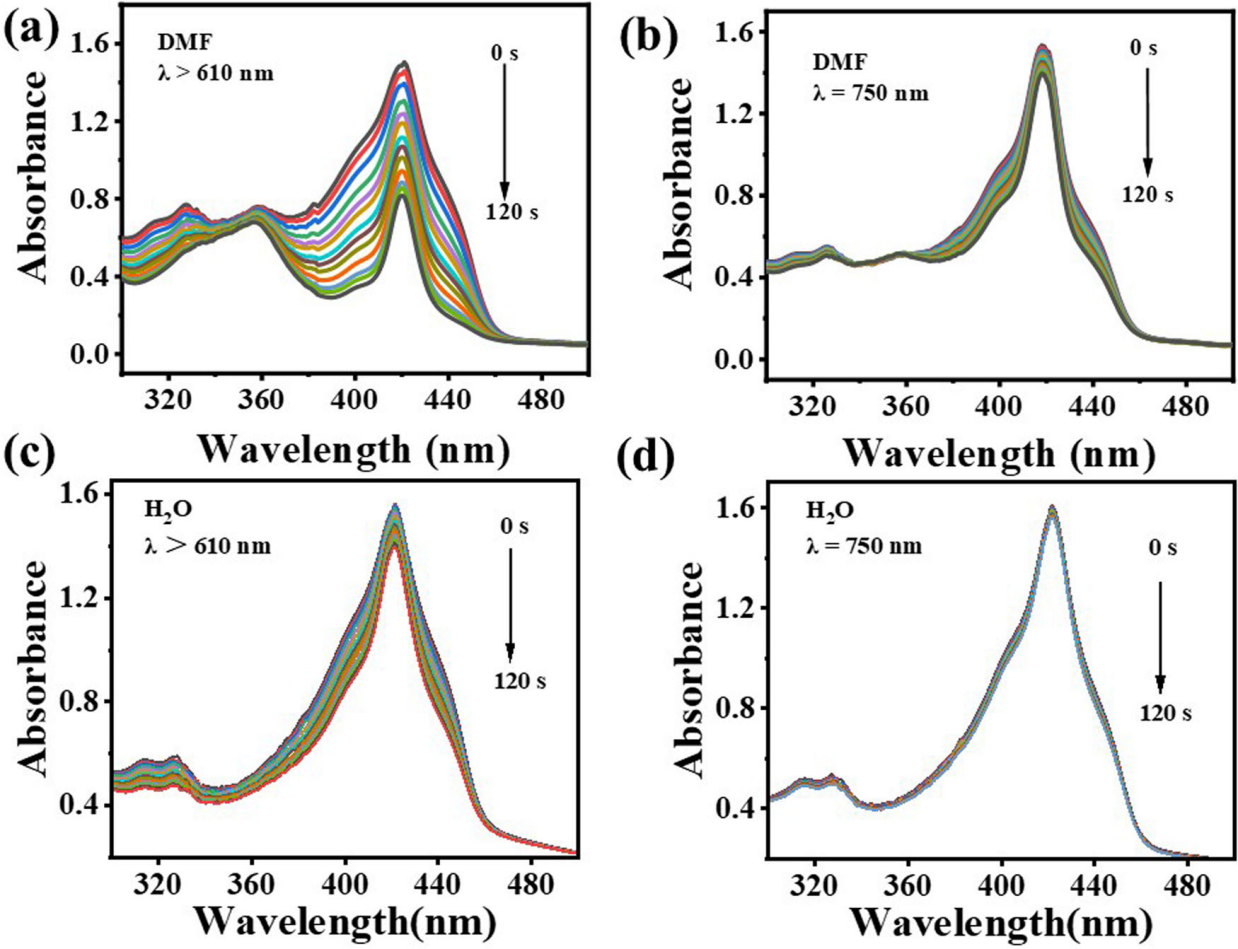
Singlet oxygen generation. UV-vis absorption spectra for the determination of ^1^O_2_ from SiPc-ddCPP irradiated by (**a**, **c**) red light (λ > 610 nm), (**b**, **d**) NIR light (λ = 750 ± 10 nm) in DMF (**a**, **b**) and water (**c**, **d**).

The effectiveness of photodynamic therapy is the capability to produce singlet oxygen (^1^O_2_). Indirect method using DPBF as ^1^O_2_ trapping agent was used to evaluate the photodynamic properties of SiPc-ddCPP. It can be seen in Fig. 6, with coexisting of SiPc-ddCPP, the absorption of DPBF at 415 nm gradually reduces along with the irradiation time by red light (*λ* > 610 nm), showing ^1^O_2_ is continually generated. For comparison, the ^1^O_2_ generation efficiencies in NIR region was also detected under irradiation by NIR light (*λ* = 750±10 nm) and it was found that ^1^O_2_ could also be generated by SiPc-ddCPP. These data clearly shows that the orthogonal structure of SiPc-ddCPP is beneficial to ^1^O_2_ generation and photodynamic therapy. The singlet oxygen yield of SiPc-ddCPP was calculated to be *Φ*_*Δ*_ = 0.17 using ZnPc as reference, which is less than half of that of SiPc-NH_2_ (*Φ*_*Δ*_ = 0.37), due to the energy exchange induced nonradiative heat relaxation caused reduced intersystem crossing pathway. In addition, type II mechanism was determined to be the main PDT pathway for SiPc-ddCPP (Supplementary Fig. 5).

### Photothermal properties of SiPc-ddCPP

**Fig. 7:**
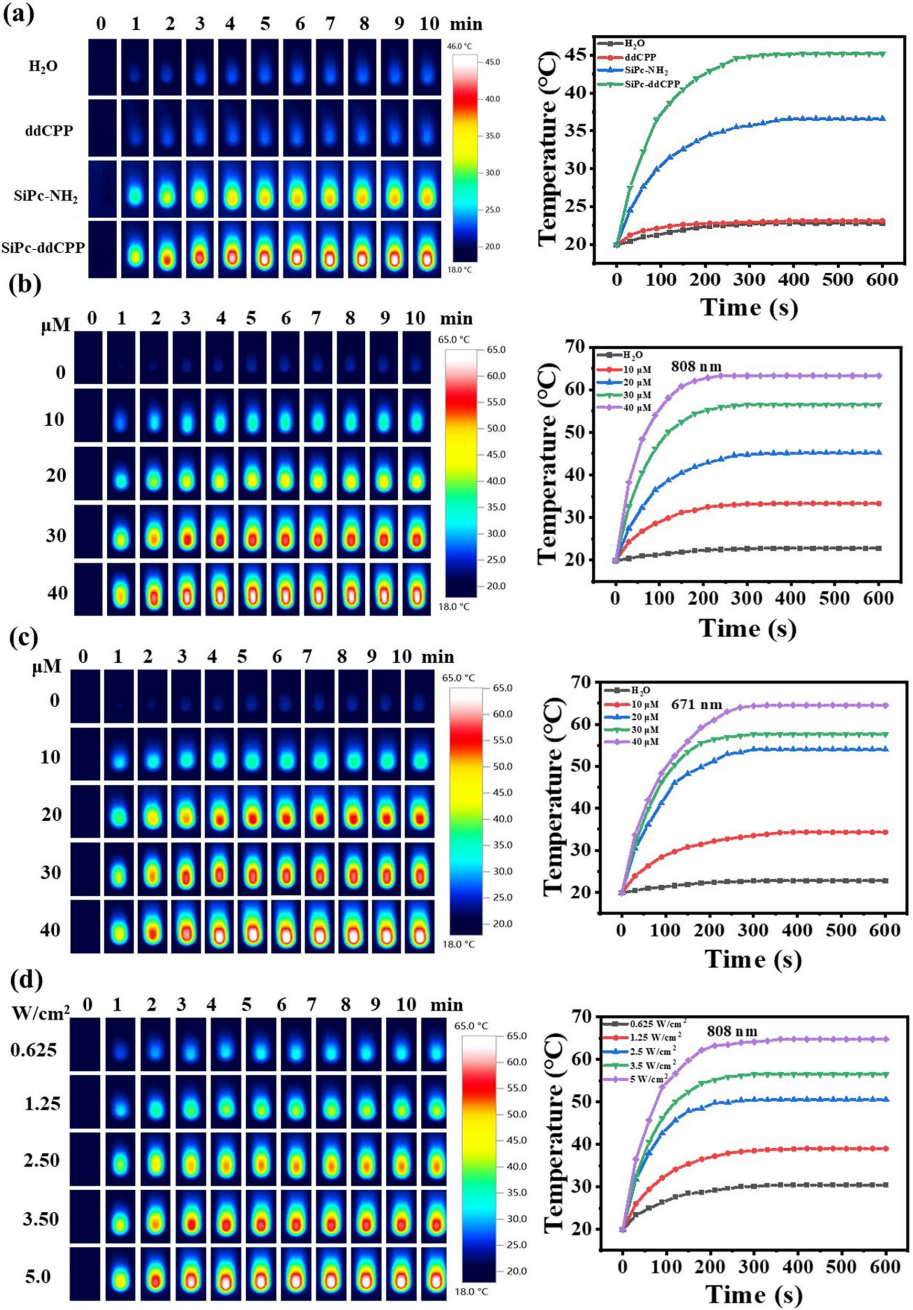
Photothermal analysis. **a** Thermal images of equivalent SiPc-NH_2_, ddCPP, SiPc-ddCPP in deionized water under 808 nm laser irradiation (3.5 W cm^-2^) and the corresponding time-dependent photothermal curves; (**b**) thermal images of SiPc-ddCPP at different concentrations under 808 nm laser irradiation (3.5 W cm^-2^) and the corresponding time-dependent photothermal curves; (**c**) thermal images of SiPc-ddCPP at different concentrations under 671 nm laser irradiation (3.5 W cm^-2^) and the corresponding time-dependent photothermal curves; (**d**) thermal images of SiPc-ddCPP (30 μM) at different 808 nm laser irradiation and the corresponding time-dependent photothermal curves.

The photothermal properties of SiPc-ddCPP was tested under both 671 nm and 808 nm laser irradiation to evaluate the photothermal effect from red to NIR region. The strong NIR absorption of SiPc-ddCPP is conducive to convert NIR light into heat for photothermal therapy. To obtain the instinct photothermal response, SiPc-NH_2_, ddCPP, and SiPc-ddCPP were compared at low concentration. Under the same conditions (808 nm), the temperature increase of SiPc-ddCPP (ΔT ≈ 25.3 °C) is much higher than that of SiPc-NH_2_ (ΔT ≈ 16.7 °C) and ddCPP (ΔT ≈ 3.2 °C), strongly certifying that SiPc-ddCPP is an excellent NIR photothermal agent. Due to the absorption absence in NIR region, the temperature of ddCPP remains almost unchanged as control H_2_O (Fig. 7a).

In contrast, SiPc-ddCPP also shows excellent photothermal response at 671 nm due to its high absorption in red region, which is comparable to the photothermal heating at 808 nm. The temperature of SiPc-ddCPP readily increase up to 65 °C under either 671 nm or 808 nm laser irradiation, exceeding the photoablation limit (50 °C) of living cells or bacteria, which convincingly show the remarkable PTT potential of SiPc-ddCPP from red to NIR region. In addition, the photothermal heating of SiPc-ddCPP was positively correlated with concentration and laser power as shown in Fig. 7. All these results exhibit that orthogonal structured SiPc-ddCPP has excellent photothermal capability for PTT from red to NIR region.

### Photothermal stability and photothermal conversion efficiency

**Fig. 8:**
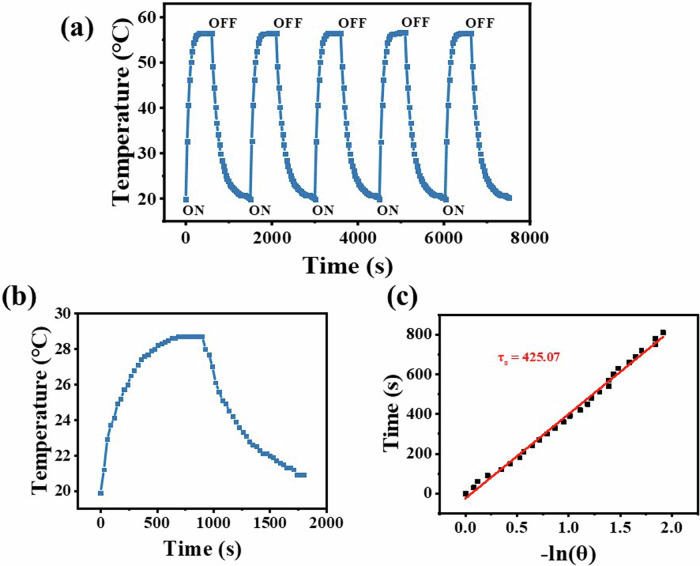
Photostability analysis. **a** The stability cycles of SiPc-ddCPP aqueous solution under 808 nm laser irradiation (3.5 Wcm^-2^); (**b**) photothermal response of SiPc–ddCPP in H_2_O under laser irradiation (808 nm, 1.25 W cm^-2^) then light shut off and (**c**) plot of the cooling time vs. the negative natural logarithm of the driving force temperature obtained from the cooling stage.

An excellent photothermal material should not only have good photothermal response and near-infrared absorption, but also have certain photostability. The photothermal stability of SiPc-ddCPP was verified by an experimental method of repeated heating/cooling cycles under high laser power irradiation (3.5 W cm^-2^). After five cycles, the heating rate and temperature increase of SiPc-ddCPP remain unchanged (Fig. 8a), which proves that SiPc-ddCPP possesses good photothermal stability and photobleaching resistance, making it a promising candidate for further clinical application. This is consistent with the good thermostability evaluated by thermogravimetric analysis as described above.

According to the published method^58^, the NIR photothermal conversion efficiency (*η*) of SiPc-ddCPP was calculated as 31.15%, which is much higher than most of inorganic and organic photothermal materials, such as Au nanorods^59^, Cu_2-x_Se nanomaterials^60^, MB^61^ and indocyanine green^62^. This efficient photothermal effect of SiPc-ddCPP in NIR region is in favor of PTT treatment of deep-seated infections. As aforementioned, both steady fluorescence and time-resolved fluorescence have successfully proved the efficient energy transfer between the two chromophores in SiPc-ddCPP. Hence, the enhanced non-radiative vibration relaxation and NIR absorption caused by intramolecular energy transfer in SiPc-ddCPP should jointly account for its remarkable photothermal conversion efficiency (Fig. 8).

### Antibacterial experiment

**Fig. 9:**
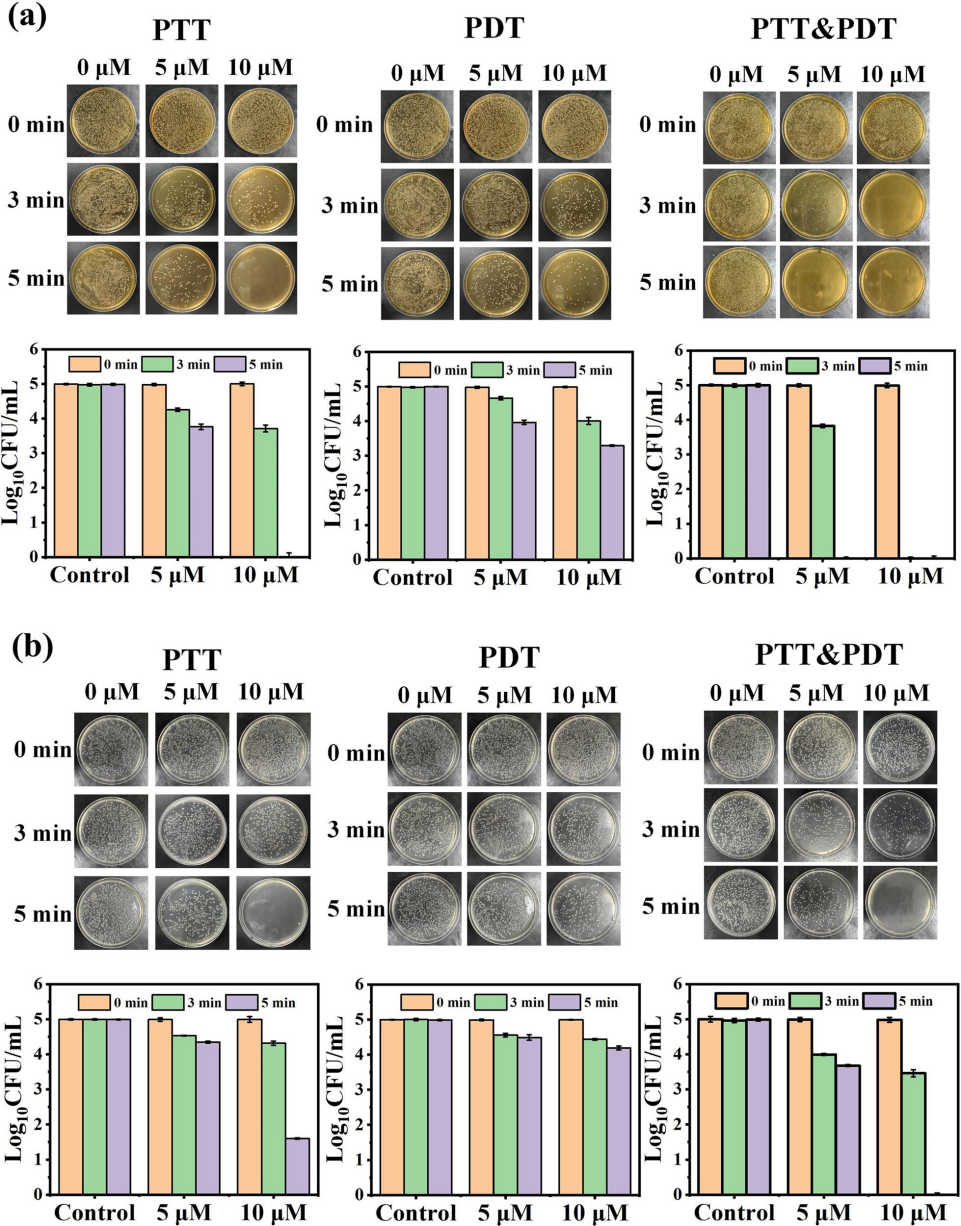
In vitro antibacterial activities. Antibacterial activity images (upper) and corresponding statistical analysis (lower) of SiPc-ddCPP against (**a**) Gram-positive bacteria (*S. aureus*) and (**b**) Gram-negative bacteria (*E. coli*) under irradiation of PTT, PDT and PTT-PDT.

**Fig. 10:**
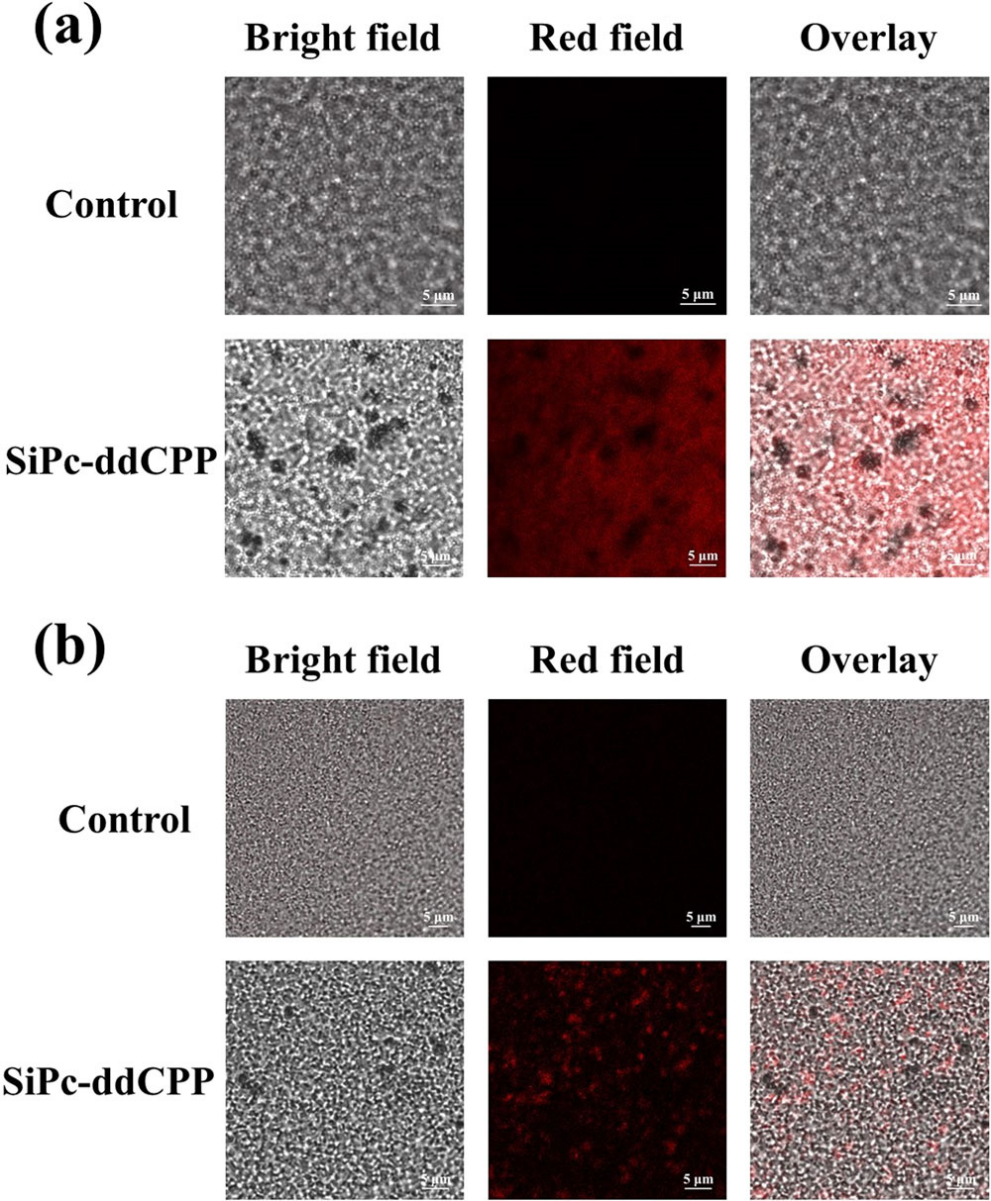
Confocal fluorescence images. **a** *S. aureus* and (**b**) *E. coli* after being incubated with medium (upper) and SiPc-ddCPP (lower).

Based on the good singlet oxygen generation and photothermal capability of SiPc-ddCPP, the PDT-PTT combined antibacterial studies were conducted on both Gram-positive bacteria (*S. aureus*) and Gram-negative anaerobic bacteria (*E. coli*). The colony counting method was used to evaluate the PDT, PTT or PDT-PTT antibacterial effect of SiPc-ddCPP. A 660 nm LED lamp and 808 nm laser were chosen for evaluated PDT and PTT efficacy, respectively. As can be seen from Fig. 9, SiPc-ddCPP is nontoxic to either *S. aureus* or *E. coli* bacteria in dark. In contrast, both S. aureus and E. coli could be efficiently inhibited under irradiation by either 660 nm LED lamp or 808 nm laser, showing its PDT and PTT antibacterial effect of SiPc-ddCPP. Due to an extra outer membrane of Gram-negative bacteria^63^, *E. coli* is less sensitive than S. aureus by either photodynamic or photothermal treatment, which is also observed by previously reported PDT/PTT antibacterial studies^64^. As expected, the combined application of PDT and PTT significantly improved the antibacterial efficacy and achieved 100% bacteria death of S. aureus and *E. coli* more easily than PDT or PTT alone (Fig. 9). Furthermore, the fluorescence of SiPc-ddCPP in *S. aureus* showed higher fluorescence intensity than that in E. coli, which was consistent with the antibacterial efficacy of SiPc-ddCPP, demonstrating that SiPc-ddCPP had a good affinity for bacteria and could be used for bacterial infections (Fig. 10).

The biocompatibility of SiPc-ddCPP was evaluated on mouse embryonic osteoblasts (MC3T3) cells (Supplementary Fig. 6). A 95% survival rate in dark and 86% survival rate post-irradiation with the concentrations up to 10 μM were observed, demonstrating the good biocompatibility of SiPc-ddCPP to normal healthy cells due to the lower uptake by normal cell than bacteria within the time for infection treatment. The octanol–water partition coefficient (*logP*) of SiPc-ddCPP also shows its good hydrophilicity (Supplementary Table 1). These results systematically exhibit that SiPc-ddCPP can be rapidly taken up by bacteria but not by normal cells. In summary, SiPc-ddCPP has significant photodynamic/photothermal synergistic antibacterial effects and can be used as bactericide for in situ treatment of bacterial infections.

## Conclusions

In summary, an orthogonal conjugated oligomer (SiPc-ddCPP) between silicon phthalocyanine (SiPc-NH_2_) and porphyrin (ddCPP) was successfully synthesized through covalent amide bond connection with uniform particle size of ~100 nm. Energy transfer from ddCPP to SiPc-NH_2_ was observed within SiPc-ddCPP as evidenced by both steady and time-resolved fluorescence. The NIR photothermal conversion efficiency (*η*) of SiPc-ddCPP was calculated to be 31.15%, along with ROS generation and fluorescence emission. Antibacterial experiments showed that SiPc-ddCPP could completely inhibit Gram-positive and Gram-negative anaerobic bacteria in a few minutes under synergistic PTT-PDT effect. Therefore, SiPc-ddCPP is a novel multifunctional NIR bactericide for PDT-PTT synergistic sterilizing of either anaerobic or aerophile bacteria for in situ treatment of bacterial infections.

## Experiments

### Chemicals and materials

5,15-diphenyl-10,20-di(4-carboxyphenyl)porphine (ddCPP) was purchased from Beijing Huawei Ruike Chemical Co., LTD. Silicon phthalocyanine dichloride (SiPcCl_2_), 2-(2-aminoethoxy)-ethanol, 1,3-diphenylisobenzofuran (DPBF), 1-(3-(dimethylamino)-propyl)-3-ethylcarbodiimide hydrochloride (EDC·HCl), N-hydroxysuccinimide (NHS), N,N-diisopropylethylamine (DIPEA) were purchased from Sigma-Aldrich. Regenerated cellulose dialysis bag (molecular weight cutoff: 1000) purchased from Shanghai Yuanye Biotechnology Co. All reactions were carried out under a nitrogen atmosphere. Pyridine, toluene and N,N-dimethylformamide were distilled from barium oxide, sodium, magnesium and barium oxide, respectively. All other reagents are of analytical grade purity and no further purification was used.

### Characterizations and instruments

Gel permeation chromatography were recorded on an Agilent 1260 Infinity II chromatograph. FT-IR spectra were acquired on a Nicolet NEXUS 670 Fourier transformation infrared (FTIR) spectrometer. The Raman spectra were recorded on a Horbin PHS-3C Raman spectrometer. X-ray-photoelectron spectroscopy (XPS) was performed on a KAlpha X-ray Photoelectron Spectrometer System (ESCALAB 250, Thermo, USA), and C 1 s (284.8 eV) was used to calibrate the peak positions for the elemental analysis. Dynamic light scattering was measured on a Zetasizer Nano ZS, Malvern Panalytical analyzer. Atomic absorption spectra were acquired on a Thermo iCE30000 spectrometer. Ultraviolet visible (UV−vis) spectra were measured on a PerkinElmer Lambda-35 UV−vis spectrometer and the samples were dispersed in H_2_O or DMF. Steady-state fluorescence spectra were obtained on a Hitachi F-2700 spectrofluorimeter equipped with a 450 W Xe lamp. The time resolved fluorescence spectra were measured using Edinburgh FLS920 spectrometer. Singlet oxygen tests were performed on a 150 W Philips QVF 133 halogen lamp. The photothermal properties were recorded by a Testo 872 infrared thermal imager. Bacterial and cell experimental data were obtained on a 30 W Boxing BS-GS-660nm LED lamp and a 4 W LSR808NL 808 nm laser.

### Synthesis

SiPc-NH_2_ was synthesized according to our previously reported procedure^56,57^. 5,15-diphenyl-10,20- di(4-carboxyphenyl)porphine (ddCPP) (21.2 mg, 0.03 mmol) was dispersed in a mixed solution of DMF/H_2_O (v/v 2:3) with ultrasonication. Then, EDC·HCl (46.0 mg, 0.24 mmol) and NHS (27.6 mg, 0.24 mmol) were added to the above solution and stirred for 3 h. Thereafter, SiPc-NH_2_ (45.0 mg, 0.06 mmol) and DIPEA (315 µL, 1.80 mmol) were added and continued to stir at room temperature under a nitrogen atmosphere for 3 days. The reaction mixture was dialyzed (molecular weight cutoff: 1000) in DMF repetitively for 2 days, and followed by dialysis in deionized water for another 2 days. Then the final product was freeze-dried to give SiPc-ddCPP as a dark powder (22.7 mg, 52.8%).

### Singlet oxygen determination

1,3-Diphenylisobenzofuran (DPBF) was used as a scavenger of the singlet oxygen (^1^O_2_). The DMF solution containing DPBF (40 μmol) and SiPc-ddCPP solution (6 μmol) was irradiated by red light (*λ* > 610 nm) or near infrared light (*λ* = 750 ± 10 nm) from a halogen lamp with corresponding filters. The absorbance of DPBF at 415 nm was recorded every 10 s along with the irradiation. To prevent DPBF from aggregation, Cremophor EL with a volume fraction of 0.1% was added into the aqueous solution.

### Photothermal effect measurement

SiPc-ddCPP was dissolved in H_2_O with sonication to different concentrations. Then, 1 mL of each solution was irradiated by a 671/808 nm laser for 10 min in a quartz cuvette. The temperature change was recorded every 30 s using an infrared thermal imager (testo 872). Equivalent amount of 5,15-diphenyl-10,20-di(4-carboxyphenyl)porphine (ddCPP) and SiPc-NH_2_ were measured under the same conditions for comparison. The laser-power dependent photothermal effect of SiPc-ddCPP were obtained by 808 nm laser irradiation with different laser power output. Photothermal conversion efficiency (*η*) of SiPc-ddCPP was measured and calculated according to reported method^58^.

### Photothermal stability

The photothermal stability of SiPc-ddCPP was assessed by repetitive heating with an 808 nm laser for 10 min, followed by natural cooling to room temperature. The photostability was evaluated according to temperature change after several cycles of high-power laser exposure (3.5 W cm^-2^).

### Antibacterial experiment

Gram-positive bacteria Staphylococcus aureus (*S. aureus*) and Gram-negative bacteria Escherichia coli (*E. coli)* were used for antibacterial experiments. The bacteria were incubated at 37 °C overnight on agar plates. Then, the bacteria stock solution was prepared at a concentration of 10^8^ bacteria/mL in saline by detecting the absorption at 600 nm (OD_600_ = 0.213 or 0.413 for S. aureus and E. coli, respectively), which were further diluted with sterilized saline to a concentration of 10^5^ bacteria per mL. Then, the bacterial solution (100 μL) was mixed with different concentrations of SiPc-ddCPP (100 μL) on a 96-well plate and immediately applied to photodynamic/photothermal inhibition test by irradiation with a 660 nm LED lamp (0.2 mW cm^-2^) or an 808 nm laser (3.0 W cm^-2^), respectively. Then, the post-irradiation mixture (50 μL) was extracted and evenly dispersed onto agar plate and incubated at 37 °C for 24 h before bacterial colony counting.

### Cytotoxicity experiment

Mouse embryonic osteoblasts (MC3T3) were utilized for cytotoxicity evaluation. Cells in 96-well plates (1 × 10^4^ per well) were incubated overnight in DMEM medium supplemented with 10% fetal bovine serum (FBS) and 1% penicillin and streptomycin (100 units mL^-1^, streptomycin 100 μg mL^-1^, respectively) at 37 °C under 5% CO_2_ for attachment. The cells were then incubated with different concentrations of SiPc-ddCPP for 24 h to detect dark-toxicity. The cells for irradiation groups incubated with SiPc-ddCPP antibacterial experiment, and then were rinsed with PBS twice and 100 μL of culture medium was added to each well before being illuminated. Then irradiation groups were exposed to 808 nm laser (3.0 W cm^-2^) or 660 nm lamp (0.2 mW cm^-2^) or their superposition for 5 min, and cell viability was assessed 24 h after irradiation using CCK-8 assay. Dark groups were acquired under the same conditions without irradiation; the control group was cultured with pure medium.

## Supplementary information

Supporting information

## Data Availability

All the data and methods are present in the main text, the supplementary materials. All of the other data supporting the findings of this study are available from the corresponding author upon reasonable request.

## References

[1] Li, B. et al. Two-dimensional antibacterial materials. *Prog. Mater. Sci.* **130**, 100976 (2022).

[2] Prothiwa, M. et al. Competitive live-cell profiling strategy for discovering inhibitors of the quinolone biosynthesis of pseudomonas aeruginosa. *J. Am. Chem. Soc.* **140**, 14019–14023 (2018). 30336005 10.1021/jacs.8b07629

[3] Wang, Z. et al. Infection microenvironment-related antibacterial nanotherapeutic strategies. *Biomaterials* **280**, 121249 (2021). 34801252 10.1016/j.biomaterials.2021.121249

[4] Mitcheltree, M. J. et al. A synthetic antibiotic class overcoming bacterial multidrug resistance. *Nature* **599**, 507–512 (2021). 34707295 10.1038/s41586-021-04045-6 PMC8549432

[5] Carrel, M. et al. USA300 methicillin-resistant staphylococcus aureus, United States, 2000–2013. *Emerg. Infect. Dis.* **21**, 1973–1980 (2015). 26484389 10.3201/eid2111.150452 PMC4622244

[6] Clatworthy, A. E. et al. Targeting virulence: a new paradigm for antimicrobial therapy. *Nat. Chem. Biol.* **3**, 541–548 (2007). 17710100 10.1038/nchembio.2007.24

[7] Dietvorst, J. et al. Current and near-future technologies for antibiotic susceptibility testing and resistant bacteria detection. *Trends Anal. Chem.* **127**, 115891 (2020).

[8] Dzuvor, C. K. O. Toward clinical applications: transforming nonantibiotic antibacterials into effective next-generation supramolecular therapeutics. *ACS Nano* **18**, 2564–2577 (2024). 38227832 10.1021/acsnano.3c11045

[9] Liu, G.-Y. et al. Antimicrobial resistance crisis: could artificial intelligence be the solution? *Military Med. Res.* **11**, 7 (2024). 10.1186/s40779-024-00510-1 PMC10804841 38254241

[10] Makvandi, P. et al. Correction to: bioengineered materials with selective antimicrobial toxicity in biomedicine. *Military Med. Res.* **10**, 30 (2023). 10.1186/s40779-023-00466-8 PMC10337126 37438838

[11] Upadhayay, A. et al. Resistance-proof antimicrobial drug discovery to combat global antimicrobial resistance threat. *Drug Resist. Updates* **66**, 100890 (2022). 10.1016/j.drup.2022.100890 36455341

[12] Wang, J. et al. Redox active Zn@MOFs as spontaneous reactive oxygen species releasing antimicrobials. *J.l Am. Chem. Soc.* **146**, 599–608 (2023). 10.1021/jacs.3c10411 38109168

[13] Cai, W. et al. Efficient antibacterial AIEgens induced ROS for selective photodynamic treatment of bacterial keratitis. *Front. Chem.* **10**, 1088935 (2022). 36688052 10.3389/fchem.2022.1088935 PMC9846558

[14] Li, W. et al. Self-actuated biomimetic nanocomposites for photothermal therapy and PD-L1 immunosuppression. *Front. Chem.* **11**, 1167586 (2023). 37007061 10.3389/fchem.2023.1167586 PMC10063802

[15] Li, X. et al. Supramolecular photosensitizers rejuvenate photodynamic therapy. *Chem. Soci. Rev.* **47**, 1174–1188 (2018). 10.1039/c7cs00594f 29334090

[16] Liu, S. et al. Two‐dimensional nanomaterials for photothermal therapy. *Angew. Chem. Int. Ed.* **59**, 5890–5900 (2020). 10.1002/anie.201911477 32017308

[17] Liu, Z. et al. Photodynamic immunotherapy of cancers based on nanotechnology: recent advances and future challenges. *J. Nanobiotechnol.* **19**, 160 (2021). 10.1186/s12951-021-00903-7 PMC8164771 34051801

[18] Song, S. et al. An NIR-II excitable AIE small molecule with multimodal phototheranostic features for orthotopic breast cancer treatment. *Adv. Mater.* **36**, 2309748 (2024). 10.1002/adma.202309748 38165653

[19] Zhi, D. et al. Photothermal therapy. *J. Control. Release.* **325**, 52–71 (2020). 32619742 10.1016/j.jconrel.2020.06.032

[20] Luo, T. et al. Nanoscale metal–organic frameworks stabilize bacteriochlorins for type I and type II photodynamic therapy. *J. Am. Chem. Soc.* **142**, 7334–7339 (2020). 32248686 10.1021/jacs.0c02129

[21] Ogilby, P. R. Singlet oxygen: there is indeed something new under the sun. *Chem. Soc. Rev.* **39**, 3181–3209 (2010). 20571680 10.1039/b926014p

[22] Wang, Y.-Y. et al. Type I photodynamic therapy by organic–inorganic hybrid materials: from strategies to applications. *Coord. Chem. Rev.* **395**, 46–62 (2019).

[23] Zou, J. et al. A phototheranostic strategy to continuously deliver singlet oxygen in the dark and hypoxic tumor microenvironment. *Angew. Chem. Int. Ed.* **59**, 8833–8838 (2020). 10.1002/anie.201914384 PMC7250713 31943602

[24] Farivar, N. et al. Pulsed photothermal therapy of solid tumors as a precondition for immunotherapy. *Small* **20**, 2309495 (2024). 10.1002/smll.202309495 38511548

[25] Liu, F. et al. Two-dimensional nanosheets with high curcumin loading content for multimodal imaging-guided combined chemo-photothermal therapy. *Biomaterials* **223**, 119470 (2019). 31526950 10.1016/j.biomaterials.2019.119470

[26] Shi, M. et al. A Golgi apparatus‐targeted photothermal agent with protein anchoring for enhanced cancer photothermal therapy. *Adv. Healthcare Mater.* **13**, 2303749 (2024). 10.1002/adhm.202303749 38483042

[27] Wang, H. et al. A dual‐targeted organic photothermal agent for enhanced photothermal therapy. *Angew. Chem. Int. Ed.* **58**, 1057–1061 (2018). 10.1002/anie.201811273 30397990

[28] Guo, Z. et al. Synthesis of BSA‐coated BiOI@Bi2S3 semiconductor heterojunction nanoparticles and their applications for radio/photodynamic/photothermal synergistic therapy of tumor. *Adv. Mater.* **29**, 1704136 (2017). 10.1002/adma.201704136 29035426

[29] Liu, G. et al. Mo2C‐derived polyoxometalate for NIR‐II photoacoustic imaging‐guided chemodynamic/photothermal synergistic therapy. *Angew. Chem. Int. Ed.* **58**, 18641–18646 (2019). 10.1002/anie.201910815 31605417

[30] Liu, H. et al. Theranostic nanomotors for tumor multimode imaging and photothermal/photodynamic synergistic therapy. *Chem. Eng. J.* **442**, 135994 (2022).

[31] Zhang, K. et al. Metal–organic framework nanoshuttle for synergistic photodynamic and low‐temperature photothermal therapy. *Adv. Funct. Mater.* **28**, 1804634 (2018).

[32] Zhang, Z. et al. An all-round athlete on the track of phototheranostics: subtly regulating the balance between radiative and nonradiative decays for multimodal imaging-guided synergistic therapy. *Adv. Mater.* **32**, 2003210 (2020). 10.1002/adma.202003210 32696561

[33] Nitzan, Y. et al. Inactivation of gram‐negative bacteria by photosensitized porphyrins. *Photochem. Photobiol.* **55**, 89–96 (2008). 10.1111/j.1751-1097.1992.tb04213.x 1534909

[34] Chen, H. et al. Gadolinium-encapsulated graphene carbon nanotheranostics for imaging-guided photodynamic therapy. *Adv. Mater.* **30**, 1802748 (2018). 10.1002/adma.201802748 PMC6435436 30035840

[35] Ma, X.-H. et al. Soluble nanographene C222: synthesis and applications for synergistic photodynamic/photothermal therapy. *J. Am. Chem. Soc.* **146**, 2411–2418 (2024). 38234111 10.1021/jacs.3c08822

[36] Guo, X. et al. Intelligent gold nanoparticles for synergistic tumor treatment via intracellular Ca2+ regulation and resulting on-demand photothermal therapy. *Chem. Eng. J.* **433**, 133850 (2021).

[37] Kesharwani, P. et al. Gold nanoparticles and gold nanorods in the landscape of cancer therapy. *Mol. Cancer* **22**, 98 (2023). 37344887 10.1186/s12943-023-01798-8 PMC10283337

[38] Liu, D. et al. HOCl-activated aggregation of gold nanoparticles for multimodality therapy of tumors. *Adv. Sci.* **8**, 2100074 (2021). 10.1002/advs.202100074 PMC8425924 34235882

[39] Huang, P. et al. AIBI modified mesoporous copper sulfide nanocomposites for efficient non‐oxygen dependent free radicals‐assisted photothermal therapy in uveal melanoma. *Small* **20**, 2312211 (2024). 10.1002/smll.202312211 38381004

[40] Nikam, A. N. et al. Copper sulphide based heterogeneous nanoplatforms for multimodal therapy and imaging of cancer: recent advances and toxicological perspectives. *Coord. Chem. Rev.* **419**, 213356 (2020).

[41] Zhang, J. et al. Beating xenograft liposarcoma using metal selenides with NIR-III photothermal ablation and bioactive selenium derivates. *Chem. Eng. J.* **481**, 148521 (2024).

[42] Guo, C. et al. A natural anthocyanin-based multifunctional theranostic agent for dual-modal imaging and photothermal anti-tumor therapy. *J. Mater. Chem. B* **9**, 7447–7460 (2021). 34551057 10.1039/d1tb00988e

[43] Guo, S. et al. Near-infrared photodynamic and photothermal co-therapy based on organic small molecular dyes. *J. Nanobiotechnol.* **21**, 348 (2023). 10.1186/s12951-023-02111-x PMC10523653 37759287

[44] Xue, P. et al. Indocyanine green-conjugated magnetic prussian blue nanoparticles for synchronous photothermal/photodynamic tumor therapy. *Nano Micro. Lett.* **10**, 74 (2018). 10.1007/s40820-018-0227-z PMC6208784 30417006

[45] Hu, H. et al. A bacteria‐responsive porphyrin for adaptable photodynamic/photothermal therapy. *Angew. Chemie.* **61**, e202200799 (2022). 10.1002/anie.202200799 35332634

[46] Yang, L. et al. Microemulsion-assisted self-assembly of indium porphyrin photosensitizers with enhanced photodynamic therapy. *ACS Nano* **18**, 3161–3172 (2024). 38227816 10.1021/acsnano.3c09399

[47] Li, X. et al. Phthalocyanine–assembled nanodots as photosensitizers for highly efficient typeI photoreactions in photodynamic therapy. *Angew. Chem. Int. Ed.* **57**, 9885–9890 (2018). 10.1002/anie.201806551 29927036

[48] Li, X. et al. Phthalocyanines as medicinal photosensitizers: developments in the last five years. *Coord. Chem. Rev.* **379**, 147–160 (2019).

[49] Zheng, B.-D. et al. Phthalocyanines as contrast agents for photothermal therapy. *Coord. Chem. Rev.* **426**, 213548 (2020).

[50] Nyman, E. S. et al. Research advances in the use of tetrapyrrolic photosensitizers for photodynamic therapy. *J. Photochem. Photobiol. B. Biol.* **73**, 1–28 (2004). 10.1016/j.jphotobiol.2003.10.002 14732247

[51] Lo, P.-C. et al. The unique features and promises of phthalocyanines as advanced photosensitisers for photodynamic therapy of cancer. *Chem. Soc. Rev.* **49**, 1041–1056 (2020). 31845688 10.1039/c9cs00129h

[52] Li, X. et al. New application of phthalocyanine molecules: from photodynamic therapy to photothermal therapy by means of structural regulation rather than formation of aggregates. *Chem. Sci.* **9**, 2098–2104 (2018). 29675251 10.1039/c7sc05115h PMC5892404

[53] Fan, S. et al. Advances and potentials of polydopamine nanosystem in photothermal-based antibacterial infection therapies. *Front. Pharmacol.* **13**, 829712 (2022). 35321326 10.3389/fphar.2022.829712 PMC8937035

[54] Fan, X.-L. et al. Magainin-modified polydopamine nanoparticles for photothermal killing of bacteria at low temperature. *Colloids Surf. B. Biointerfaces* **183**, 110423 (2019). 31437608 10.1016/j.colsurfb.2019.110423

[55] Wu, F. et al. Intriguing H-aggregates of heptamethine cyanine for imaging-guided photothermal cancer therapy. *ACS Appl. Mater. Interfaces* **12**, 32388–32396 (2020). 32597630 10.1021/acsami.0c07608

[56] Ouyang, A. et al. Covalent RGD–graphene–phthalocyanine nanocomposite for fluorescence imaging-guided dual active/passive tumor-targeted combinatorial phototherapy. *J. Mater. Chem. B* **10**, 306–320 (2021). 10.1039/d1tb02254g 34935023

[57] Pan, J. et al. Fluorescent phthalocyanine–graphene conjugate with enhanced NIR absorbance for imaging and multi-modality therapy. *ACS Appl. Nano Mater.* **1**, 2785–2795 (2018).

[58] Zhu, X. et al. Temperature-feedback upconversion nanocomposite for accurate photothermal therapy at facile temperature. *Nat. Commun.* **7**, 10437 (2016). 26842674 10.1038/ncomms10437 PMC4742858

[59] Tian, Q. et al. Hydrophilic Cu_9_S_5_ nanocrystals: a photothermal agent with a 25.7% heat conversion efficiency for photothermal ablation of cancer cells in vivo. *ACS Nano* **5**, 9761–9771 (2011). 22059851 10.1021/nn203293t

[60] Hessel, C. M. et al. Copper selenide nanocrystals for photothermal therapy. *Nano Lett* **11**, 2560–2566 (2011). 21553924 10.1021/nl201400z PMC3111000

[61] Fan, Z. et al. Enhancing targeted tumor treatment by near IR light-activatable photodynamic–photothermal synergistic therapy. *Mol. Pharm.* **11**, 1109–1116 (2014). 24568338 10.1021/mp4002816 PMC3983349

[62] Jiang, Y. et al. Indocyanine green derived carbon dots with significantly enhanced properties for efficient photothermal therapy. *Nanoscale* **15**, 1925–1936 (2023). 36625142 10.1039/d2nr06058b

[63] Banfi, S. et al. Antibacterial activity of tetraaryl-porphyrin photosensitizers: an in vitro study on gram negative and gram positive bacteria. *J. Photochem. Photobiol. B. Biol.* **85**, 28–38 (2006). 10.1016/j.jphotobiol.2006.04.003 16737820

[64] Nyamu, S. N. et al. Antimicrobial photodynamic activity of phthalocyanine derivatives. *Adv. Chem.* **2018**, 1–8 (2018).

