# Supplementary material for: Orthogonally conjugated phthalocyanine-porphyrin oligomer for NIR photothermal-photodynamic antibacterial treatment
Source: Commun Chem. 2025 Mar 14;8:80. doi: 10.1038/s42004-025-01470-w (PMC11909192; doi:10.1038/s42004-025-01470-w)
Supplement: Supplementary file 1 — Supporting information [file 42004_2025_1470_MOESM1_ESM.pdf]

SUPPORTING INFORMATION

Supplementary Figure 1: Schematic illustration

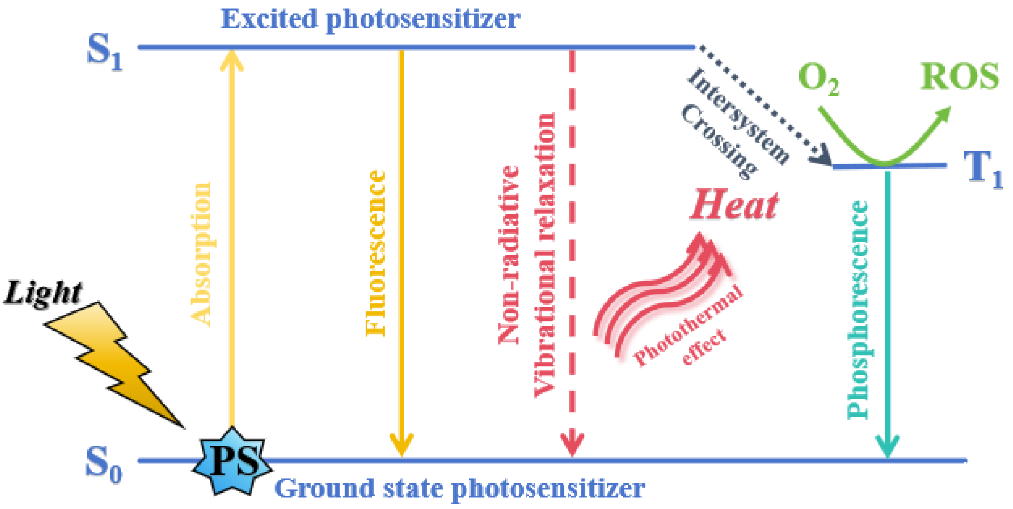

Simplified Jablonski energy level diagram.

Supplementary Figure 2: Si content

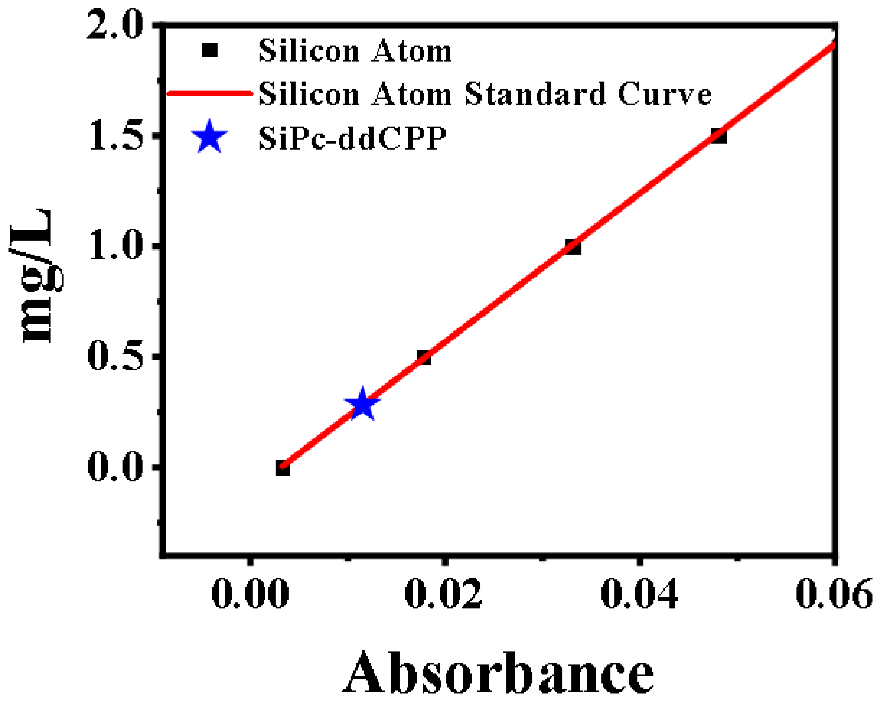

AAS of SiPc-ddCPP in DMF.

Supplementary Figure 3: Fluorescence.

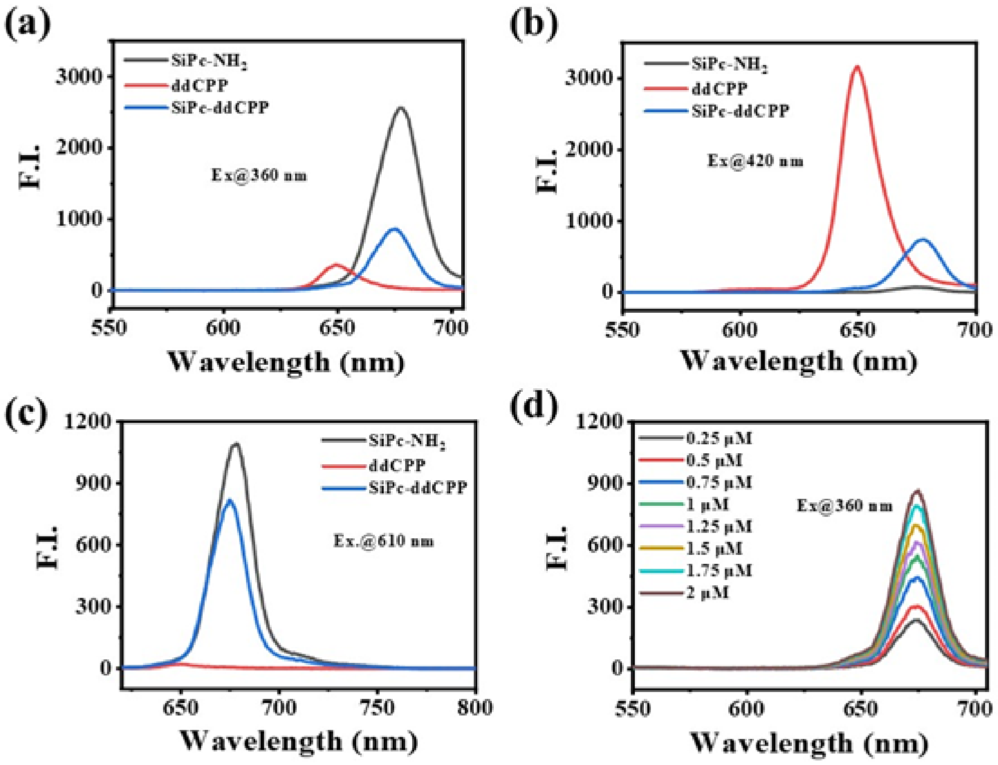

Fluorescence spectra of equivalent SiPc-NH_2, ddCPP and SiPc-ddCPP excited at (a) 360 nm, (b) 420 nm and (c) 610 nm in DMF; (d) Fluorescence spectra of SiPc-ddCPP at different concentrations excited at 360 nm in DMF.

Supplementary Figure 4: Time-resolved fluorescence

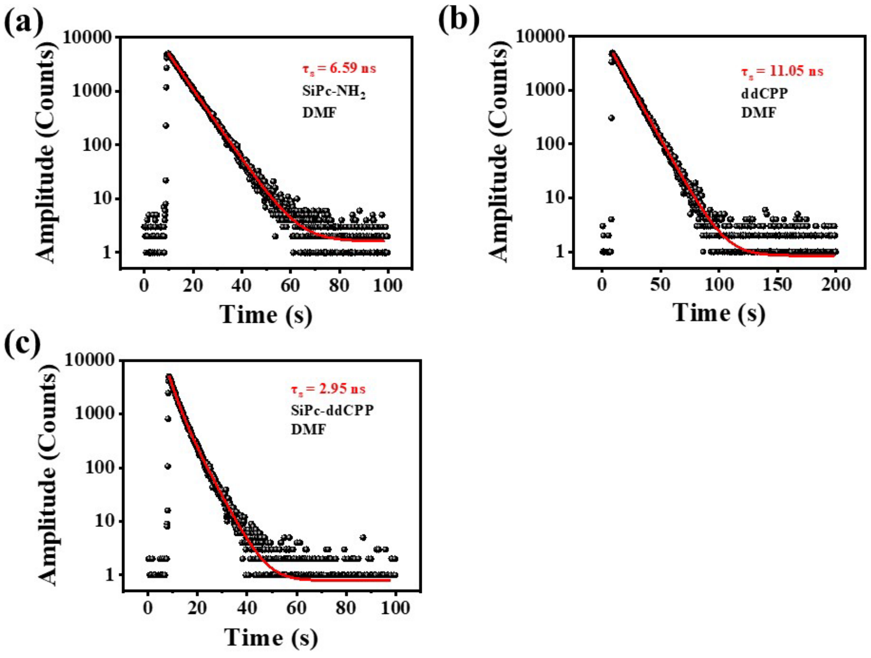

(a) SiPc-NH_2, (b) ddCPP and (c) SiPc-ddCPP in DMF.

Supplementary Figure 5: PDT mechanism

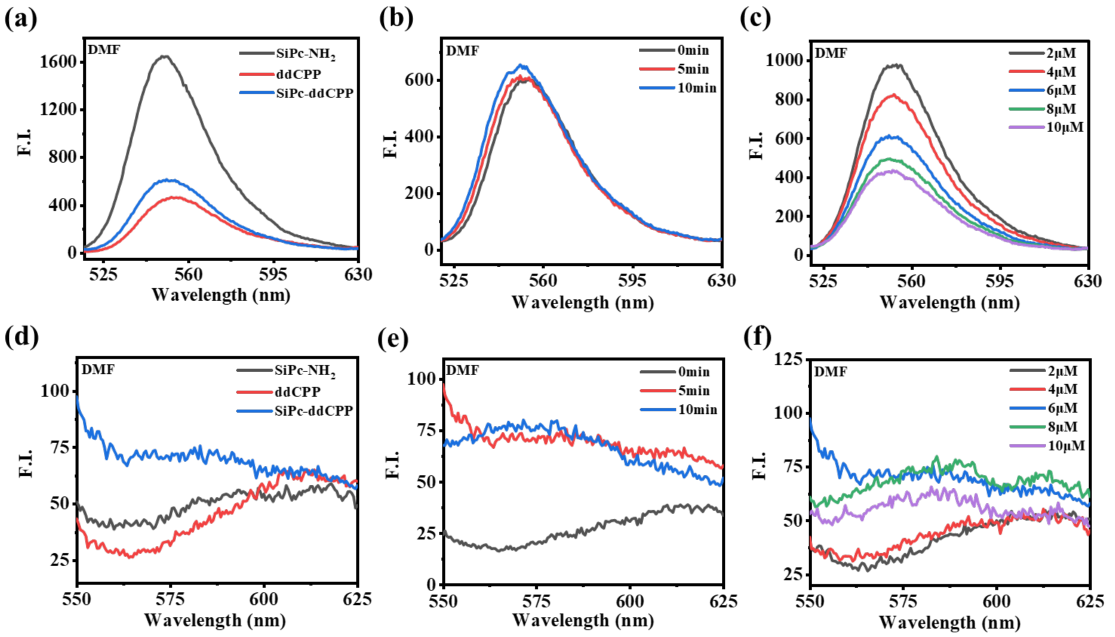

Fluorescence intensity for detection $^1O_2$ generation (a) at equivalent of SiPc-NH2, ddCPP and SiPc-ddCPP after irradiating 5 min (b) at different irradiating time (c) at different concentration of SiPc-ddCPP using SOSG probe; Fluorescence intensity for detection $O_2^{\bullet -}$ (d) at equivalent of SiPc-NH_2, ddCPP and SiPc-ddCPP after irradiating 5 min (e) at different irradiating time (f) at different concentration of SiPc-ddCPP using DHE probe.

Supplementary Figure 6: Biocompatibility

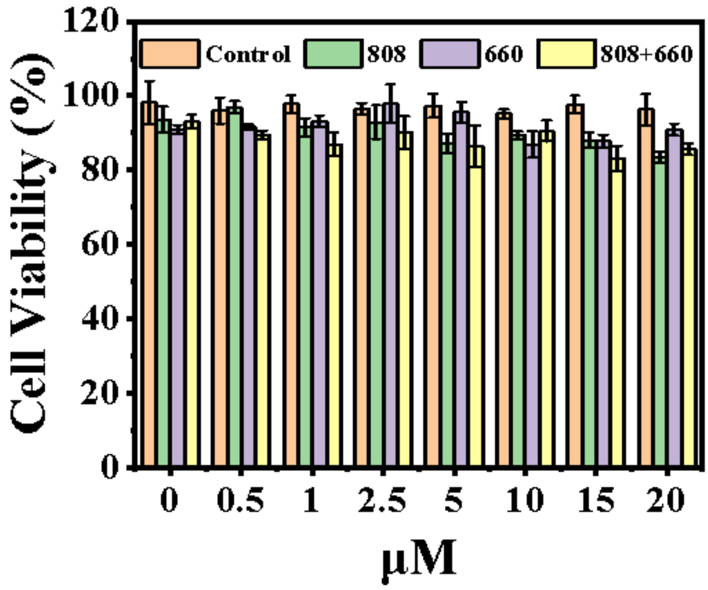

Cytotoxicity of MC3T3 cells treated with SiPc-ddCPP at different concentrations and irradiated by different light powers (808 nm laser at 3.0 W/cm^2 for PTT, LED light λ = 660 nm 0.2 mW/cm^2 for PDT) for 5 min.

Supplementary Table 1: Hydrophilicity

	SiPc-ddCPP	SiPc-NH_2	ddCPP
Log P	0.8485	0.9519	0.2821

The octanol–water partition coefficient (logP) of compounds.
